# Intelligence

**Published:** 1830-03

**Authors:** 


### INTELLIGENCE.

### COLLEGE OF PHYSICIANS.

THE evening meetings held at the College of Physicians commenced on Monday, February 8th, under the most auspicious circumstances. Among the company present were the Duke of Wellington, Lord Lyndhurst, Lord Tenterden, Lord Westmoreland, Lord Stanhope, several bishops, judges, and other distinguished persons. The presence of so many noblemen and members of the government gave great eclat to the converzatione; and their honouring the College on this occasion is calculated to raise our profession in public estimation, by showing how highly it is appreciated by men of the highest rank, and holding the most prominent stations in the country. When we reflect how industriously it has been attempted by some to lower us in the public eye, we feel doubly called upon to express our sense of the obligation under which we lie to Sir Henry Halford, to whose connexion with the aristocracy, and to whose zeal in promoting the honour and dignity of the profession over which he so ably presides, the College is indebted for the presence of these distinguished visitors.

The learned President having taken the chair, with the Lord Chancellor on his right and the First Lord of the Treasury on his left, congratulated the meeting on the advantages which they had gained from the establishment of statistical reports on the nature of disease as it presents itself in foreign countries; and he spoke in high terms of the facilities which had been afforded in effecting this purpose by various members of his Majesty's government.

After these preliminary remarks, Sir Henry proceeded to lay before the meeting an interesting paper, which turned chiefly on the prophetic power attributed by so many of the philosophers and poets of antiquity to the last moments of life; a subject calculated to arrest attention; while additional interest was excited by the elegant language in which the essay was written, by the numerous classical illustrations in which it abounded, and by the emphatic and excellent manner in which it was read by the learned and accomplished author.

### Sir HENRY HALFORD *on the Prophetic Power said to occur before Death in the* Καυσος, *or Brain Fever.*

Sir Henry began by observing, that he regarded the description given by Aretæus of the καυσος, or burning fever of Hippocrates, (the brain fever of English authors,) as one of the most interesting specimens of medical literature which had come down to us from antiquity; remarkable alike for the beauty of the language (Ionic Greek,) and the fidelity of the details. It was not to the early stage, similar in its phenomena to other inflammatory diseases, but to its termination, that the author was anxious to direct attention; a termination ushered in by syncope, followed by cold sweats, and "a loosening of all the bonds by which the human frame is held together."

Aretæus represents the patient as the first to discover his approaching end, and announcing it to his attendants; as seeming to hold converse with those gone before him, and acquiring a prophetic power in the last moments of existence; while he attempts to account for this by supposing that the soul, whilst "shifting off this mortal coil," becomes purer and more spiritual, as if its new existence had already commenced.

This account of the description of Aretæus was followed by the history of a case which had fallen under the care of the learned President. A young gentleman, who had been using mercury, caught cold while under its influence, and became affected with fever. On the seventh day, when Sir Henry was first called in, he was in a state of the highest excitement; threatening those around him, and not to be approached without increasing his irritation to fury. He was put under restraint, and tartarized antimony administered at intervals, in doses of a grain each time. On the eleventh day from the commencement of the attack, he became quite calm, and to those about him he seemed much better. It was observed that he had repeatedly said he should die, and had talked with the utmost composure of his affairs, giving directions for their arrangement. He sent messages to his absent friends, and spoke of a sister recently dead, as one whom he was about immediately to follow. In answer to his interrogations, Sir Henry found that he had not slept anterior to this quietude, and that his pulse was quicker than ever. He then became satisfied that the improvement was but in appearance, that it was "a lightning before death," and that the hours of his patient were numbered. He died that night.

The author then alluded to the case which he had related last year, in which a gentleman, labouring under insanity, was put to Shakspeare's test of "re-wording" his meaning. In this case, also, some restoration of the mind took place before death; but, as the case was a chronic one, the phenomena were different: different as delirium from insanity. The mention of this distinction led the author to allude to the eloquent pleading of Lord Erskine, in defence of Hatfield, who was tried for shooting at the King. "In some cases," said he, "perhaps in several, the human mind is stormed in its citadel, and laid prostrate under the stroke of frenzy. These unhappy sufferers, however, are not considered by physicians as maniacs, but to be in a state of delirium from fever. There, indeed, all the ideas are overwhelmed, for reason is not merely disturbed, but wholly driven from her seat. In others, reason is not driven from her seat, but distraction sits down upon it along with her, holds her trembling upon it, and frightens her from her propriety."

Returning to Aretæus, and the prophetic power attributed by him to patients under this form of fever, the learned President observed that it did not appear to him necessary to attribute the phenomena to any supernatural influence. We were accustomed to see the mind frequently "clear up" in the last hours of life, especially when this is cut short by diseases which have previously disturbed the intellectual faculties. The mind becoming capable of exercising the most correct judgment when no longer biassed by the passions, and the experience of the past giving wisdom to the inferences as to the future; such being a period, according to the lines of Milton,

"When old experience does attain
To something of prophetic strain."

The author next entered into a curious and erudite discussion, in which he displayed great ingenuity and research. The object was to prove, by numerous illustrations, the general prevalence in ancient times of a belief that some prophetic power attended the last hours of existence. He began by referring to holy writ, quoting the passage from the Pentateuch, where it is said that

"when Jacob had made an end of commanding his sons, he drew up his feet into the bed, and yielded up the ghost." The former part of this passage, Sir Henry thought, might be more faithfully rendered, "when Jacob had finished imparting his solemn injunctions to his sons;" injunctions which were mixed up with much prophetic matter. And, although the learned President, believing the narrative of Moses to have been guided by the light of inspiration, and therefore not to be humiliated by being compared even with the sublime description of disease to which he alluded, still, he observed, it was remarkable that the Deity should think fit to choose the dying hour of the patriarch in which to enlighten his mind as to his gracious purposes for the future.

The fame of Jacob's prophecy, as well as of those of Isaiah, extended far beyond the limits of the country in which they were made; and the learned President deemed it probable that they had spread over the whole of the Roman empire by the authority of the Sibylline leaves. The general belief which attributed the gift of prophecy to the hour of death is alluded to by many, both of the Greek and Roman authors; and among others, Cicero, no less distinguished as an orator than as a philosopher, in his first book "De Divinatione," mentions that the death of Alexander the Great had been predicted by an Indian about to die on the pile. In the sixteenth book of the Iliad, Patroclus foretells the death of Hector; while Hector, in his last moments, prophesies the fall of Achilles by the hand of Paris.

The same idea of prophetic power is seen in Virgil, who makes Orodes (tenth book of Æneid) predict the death of Mezentius, by whom he had just been mortally wounded.

> "Non me, quicunque es, inulto,

> Victor, nec longûm lætabere: te quoquè fata

> Prospectant paria, atque eadem mox arva tenebis."

So also Shakspeare, when Hotspur falls in combat with Harry Monmouth,

> "Oh! I could prophesy,
> But that the earthy and cold hand of death
> Lies on my tongue."

In Richard the Second, too, we find John of Gaunt, when dying, exclaim,

> "Methinks I am a prophet new inspired."

The author concluded nearly in these words: "I have extended this speculative part of my paper to too great a length: not that I dread the reproach of those among you who delight to mix the elegancies of literature with the severer studies of your profession; nor do I fear the disapprobation of others who are intent only on acquiring a knowledge of physic; they will surely thank me for having laid before them so faithful, so beautiful an historian of disease as Aretæus."

---

### REGULATIONS WITH REGARD TO PAPERS.

*Resolutions of the Committee appointed to receive and consider the Papers presented to the College.*

1. All papers proposed to be read at the evening meetings of the College, should be sent to the registrar at the College of Physicians, who will acknowledge the receipt of them by a nôtice to their respective authors.

2. All papers thus received will be laid before the president and committee, who will arrange the order in which they shall be read.

3. All papers will be read to the meeting by the registrar, or his deputy, in the presence of the president or pro-president.

4. Notice will be given to each author of the evening on which his paper will be read.

5. At the end of each year, a selection of such papers as may be deemed useful for publication, whether read or not at the College, will be made, and, with the consent of the authors, printed in the Transactions.

6. Such papers as either, from want of time, may not be read at the College, or not deemed desirable for publication, will be returned to the authors at their request.

7. The reading of papers will commence at a quarter-past nine o'clock precisely, and will not be protracted beyond ten o'clock.

8. No paper which has been previously read before any other Society will be admissible.—*Med. Gazette.*

### ROYAL INSTITUTION.

*Natural History of the Oak.* Mr. Gilbert Burnett recently delivered a very interesting lecture upon the natural history of the oak. Mr. Burnett observed, that much confusion had existed as to the botanical names and the number of species of our native oaks. Linnæus considered them all varieties of one species, which he named *Quercus robur*. Later botanists distinguished several species. He was inclined to think there were three, and only three, indigenous oak; viz. the stalk-fruited, the sessile-fruited, and the downy: and that the others are mere varieties, or probably mule plants, formed by the mutual impregnation of the three above-named species. Smith calls the first Q. robur; Willdenow applies the specific name, robur, to the second; while Mr. B. argued that the characters ascribed by the ancients to their robur agreed the most closely with the third. With regard to the acorn, formerly so much used as an article of food, Mr. Burnett stated that they were rendered much more palatable, as well as more nutritious, by allowing them to germinate, and then suddenly checking their growth by heat, as in malting. A similar plan might be followed with the horsechesnut, which might thus be rendered fit for service. The mode by which this process acts is by the formation of a considerable portion of sugar.

Some acorn bread and biscuits were exhibited, of which we anxiously sought to procure a taste; but so keen was the appetite of the naturalists present, or so great their curiosity to determine whether the ordinary food of their forefathers could be again brought into use, that we retired unsuccessful, and without a single crumb. Mr. Burnett's discourse was much and deservedly applauded.

### COLLEGE OF SURGEONS.

*Hunterian Oration.* The last Hunterian Oration was delivered by Mr. Guthrie, to a very numerous audience. We have not room to enter into the subject of it, but we must observe, that it was correctly conceived and eloquently delivered. They who most delight in censure have admitted that Mr. Guthrie showed his superiority as a public speaker over most of those who have appeared in the character of annual orators at the College of Surgeons.

*Exposure of M. Chabert, the* soi-disant *Poison Swallower and "Fire King."*

We have hitherto refrained from making any observations respecting the feats of this personage. Like many others, he has dealt largely in promises, and but scantily in performance. His exhibition of remaining in an oven heated to a very high temperature, while meat was roasted, was not calculated to attract much curiosity. Neither was there any thing marvellous in his swallowing boiling oil: such feats as these have often been performed before, by some from their love of science, by others as an exhibition for money. But when M. Chabert asserted that he could swallow poisons with perfect safety, in consequence of possessing a certain antidote to their destructive effects, the curiosity of the public, and the scientific and medical part of it especially, was instantly roused. We had before some reasons to believe he was a rank impostor, but now he is proved to be so beyond the possibility of doubt. Mr. WAKLEY lately put his pretensions to the test, by requesting him to perform his promise of swallowing prussic acid. M. Chabert positively refused, but offered to give it to some dogs! Upon this his audience became unruly, and indignant at having been duped out of their money; and the impostor made his escape as quickly as possible. It is not for us to describe the confusion that followed. We wish only to record the fact, that an antidote for prussic acid, arsenic, &c. is yet to be obtained, for M. Chabert is in possession of no such valuable agent.

### MEDICO-BOTANICAL SOCIETY. (*Circular.*)

The Council of the Medico-Botanical Society of London considers it to be its duty to inform those who in this and in other countries belong to the Society, or may wish to communicate with it,

That all communications intended for the Society are to be addressed to the president, Earl STANHOPE, No. 12, Albemarle street, London; or to one of the secretaries, Dr. SIGMOND, No. 24, Dover street, Piccadilly, and HUMPHREY GIBBS, Esq. No. 47, Half-moon street, Piccadilly, London.

That all payments to the Society are to be made to the treasurer, THOMAS GIBBS, Esq. No. 47, Half-moon street, Piccadilly, London.

And that Mr. JOHN FROST, who was formerly the Director of the Society, has, by the Council and by the Society, been suspended from the functions of that office; and that the office of director now no longer exists.

By order of the Council,

STANHOPE, *President.*

### MEDICAL SCHOOL, ALDERSGATE STREET.

The third anniversary festival of this Institution took place on Friday, the 19th instant, at the London Coffeehouse, Ludgate hill; Mr. TYRRELL in the chair. About one hundred gentlemen (chiefly those who have been educated at the School) sat down to an excellent dinner, and the utmost hilarity prevailed throughout the evening. Judging from the number present, and the enthusiasm with which the healths of the different teachers were drank, we are inclined to augur favorably of the establishment. According to the statement of the chairman, the past season has been more prosperous than either of the preceding.

### LITERARY NOTICE.

Dr. CALVERT HOLLAND has in the press a work entitled "The Physiology of the Fœtus, the Liver, and the Spleen."

### THE MEDICAL PROVIDENT INSTITUTION OF SCOTLAND,

*For the granting of Benefits during Professional Incapacity; and, in old age, Annuities to Widows, or others.*

Although societies have existed for a considerable period both in Scotland and England, in which men of every profession could make provision for old age, and for their families or other dependents, after death—it has excited the surprize of many, that, until the formation of the Medical Provident Institution of Scotland, there was no fund established by any other society, with the exception of the funds of Friendly Societies, among the labouring classes, by contributing to which, provision could be made for a day of sickness.

The objects of this institution are generally—To protect the members throughout their whole lives, and to make provision for their widows, children, or other dependents, after their death. The casualties to which professional men are exposed, are—First, occasional or temporary inability to continue the personal exertions upon which their incomes depend, by reason of sickness or accident; and, second, The more durable or permanent incapacity arising from the infirmities of old age.

The scheme embraces the following benefits:—I. Health Assurance—II. Deferred Annuities, and III. Contingent Annuities.

I. *Health Assurance.*—Under this head, provision is made for professional incapacity, whether arising from sickness or accident, and this is combined with a permanent annuity in old age.

II. *Deferred Annuities*—or annuities in old age, form the second table in the scheme. These annuities are combined with the health assurance in the former table; but they may also be assured separately, and may be entered upon at 50, 55, or 60: there is also a payment equal to a whole year's annuity, within three months after death.

III. *Contingent Annuities*—or annuities to widows or other survivors, form the third table. These annuities, contingent on the wife surviving the husband, are too well known to require any description here. They may be granted to others than the wives of members, such as brothers, sisters, or other nominees.

The Medical Provident Institution is a *mutual* assurance scheme, and the whole funds belong to the assured; and, should the rates of contribution be found to be higher than they might have been, the surplus will, in one form or other, be made available to the members, and not carried off by any body of proprietors.

Forms of the proposals, and certificates, and every necessary information, may be obtained on application to Mr. D. Cannan, Secretaiy, 41, Northumberland street, Edinburgh.

---

Dr. John Brown, *the Author of the Theory of Medicine.* It is well known that this admirable scholar, and most ingenious medical theorist, died at a comparatively early age, leaving a wife and young family entirely unprovided for. Had he lived longer, he would have enjoyed the gratification of knowing that his doctrines were adopted on the continent of Europe, particularly in Germany and Italy. Indeed they form the basis of the more recent theory of Rasori and Tommasini, which is now very generally adopted in the latter country. Of Dr. Brown's family, a son and daughter are still alive; the son held the appointment of purser in the navy, but has now to provide for a family with a pittance of half-pay. The daughter has supported herself respectably through life, as a private governess; but age and infirmity have overtaken her, and she is now entirely unprovided for. She is desirous of publishing, by subscription, a work of imagination; and, as the inquiries we have made respecting her have afforded us the utmost satisfaction, we make this appeal to the profession in her behalf. The names of Subscribers will be received at our Publisher's, or at any Medical Bookseller's. We are happy to add, that the most eminent medical men in the metropolis have, with a liberal feeling, subscribed to her undertaking.

---

*Sale of the Remainder of* Mr. BROOKES's *Museum.* The remainder of this invaluable Anatomical and Zootomical Museum is to be sold by auction by Messrs. WHEATLEY and ADLARD. The first day of the sale will be Monday, March 1st, at half-past six in the evening, at the Theatre of Anatomy, Great Marlborough street. Such an opportunity of purchasing rare examples of natural history, and natural and morbid anatomy, may never again occur, and will doubtless attract a numerous and scientific company.

